# Factors promoting the natural regeneration of *Larix principis-rupprechtii* plantation in the Lvliang Mountains of central China

**DOI:** 10.7717/peerj.9339

**Published:** 2020-06-17

**Authors:** Wenjun Liang, Xi Wei

**Affiliations:** College of Forestry, Shanxi Agricultural University, Taigu, China

**Keywords:** Forest plantations, Natural regeneration, Environmental factors, Forest structure, Soil nutrients

## Abstract

Given their complexity, targeted care and management of different areas and tree species are necessary for enhancing the natural regeneration of forests. Thus, an understanding of changes in the overstory and understory is essential for ensuring successful regeneration. Promoting the natural regeneration of *Larix principis-rupprechtii* plantations is considered challenging; indeed, regional sustainable development through natural regeneration of many stands has often been considered unattainable. Here, we studied several plots with varying extents of regeneration to identify the most important factors that affect regeneration. The plots were divided into three forest types based on the number of regenerating plants. For each type of plot, we measured various factors that might potentially affect regeneration. Representational difference analysis was used to identify the most important factors >9% contribution). Based on these factors, multiple corrections were made to construct a structural equation model of topography, stand structure, soil properties and litter to identify the most important factors driving variation in regeneration. Positive correlations were detected between regeneration with diameter at breast height (0.21) and litter thickness (0.57). Regeneration was negatively correlated with soil (−0.54) and slope (−0.48). Additionally, the number of regenerating plants and the height of regenerating plants were strongly positively correlated. However, there was no significant relationship between regeneration and litter accumulation, stand density, altitude, average tree height, total P and total N. Overall, our study showed that key factors for promoting natural regeneration include appropriate litter thickness, strong parent trees, a gentle slope and sufficient quantities of soil nutrients. Moreover, our findings provide a reference for the design of effective management and restoration plans.

## Introduction

Forest regeneration provides the next generation of overstory trees, which occupy an important position in forest ecosystems (Abe et al., 2008), and is considered one of the most vital processes in the replacement of old trees with young ones (*Singh, Malik & Sharma, 2016*). Natural regeneration ensures the spatial and temporal continuity of forest cover and the stability of the ecological and social benefits provided by forest ecosystems (*Puhlick, Laughlin & Moore, 2012; Chen & Cao, 2014*). Over the past several decades, there has been increased interest in the use of natural forest management (i.e., “close-to-nature management”) as a preferred regeneration method over traditional planting (*Puettmann et al., 2015; Ammer et al., 2018*). Natural regeneration provides many advantages relative to artificial regeneration: namely lower costs, superior adaptation to microhabitats, and higher seedling densities (*Kolo, Ankerst & Knoke, 2017*).

North China larch (*Larix principis-rupprechtii*) is an endemic and dominant tree species in northern China, where it is often found in pure stands. Identification of the factors controlling the natural regeneration of trees is a central goal in forest management. Natural regeneration in various forest ecosystems is dependent on several abiotic and biotic factors that either directly or indirectly influence the success of regeneration (*Rooney, Solheim & Waller, 2002; Krauss & Allen, 2003; Noguchi & Yoshida, 2004*). Thus, a deeper understanding of the fundamental properties underlying tree regeneration is necessary for ensuring that afforestation practices are capable of adapting to future abiotic and biotic conditions.

Generally, the main factors affecting regeneration in a stand include stand density, the quality of seed trees (Shepperd, Edminster & Mata, 2006; Muhamed et al., 2018; Ali et al., 2019) and the spatial configuration of overstory trees (Sánchez Meador et al., 2009). Seedlings are an important stage during natural vegetation restoration (*Sansevero et al., 2017*). The relative proportions of different age groups of species ultimately determine the breeding population and the long-term development of forest ecosystems (*Soucy, Lussier & Lavoie, 2012; Sharma et al., 2016*). Sufficient numbers of young plants, seedlings and saplings are essential for regeneration; in contrast, insufficient numbers are indicative of poor regeneration ability (*Ali et al., 2019*). Factors that can affect the success of young plants, seedlings and saplings by regulating seed germination, the initial development of seedlings and the composition and abundance of competitive vegetation include soil temperature, nutrient mineralization, light availability in the understory and stand structure (*Puhlick, Laughlin & Moore, 2012; Herizo, Randriamalala & Carrière, 2019*). For example, study of stand age structure and spatial patterns has provided critical insight into the role of small-scale processes and the interaction between canopy cover and regeneration establishment in temperature-limited environments (*Carrer, Soraruf & Lingua, 2013*). However, soil characteristics, such as pH, organic matter and other nutrient indices, also influence patterns of regeneration through their effect on the production and composition of trees, shrubs and herbs, especially the herbaceous understory (*Abella & Covington, 2006; Laughlin et al., 2007*).

Here, we assessed the relative importance of direct and indirect factors affecting the regeneration of *L. principis-rupprechtii* plantations. Inventory data from previous studies have indicated that temperature stress and moisture availability play more important roles in Mediterranean forest relative to temperate forest (*Bravo et al., 2008; Vayreda et al., 2013; Monteiro-henriques & Fernandes, 2018*). Other factors have comparatively minor effects on tree regeneration; however, few studies have comprehensively examined the relative roles of these factors on tree regeneration in both the understory and overstory layers (*Plieninger, Rolo & Moreno, 2010; Shen & Nelson, 2018*). Furthermore, these studies have generally not explored how the structure of the overstory and the understory might interact to affect regeneration (*Lombaerde et al., 2019*).

Identifying the factors and the complex interactions that affect the regeneration of *L. principis-rupprechtii* plantations is critical for planning and forest management activities (*Puhlick, Laughlin & Moore, 2012*). These factors are ultimately related to patterns of natural disturbance and the genetic characteristics of the forests; thus, elucidating the role that these factors play would provide a foundation for which the possible outcomes of different silvicultural measures can be assessed. To ensure that regeneration proceeds successfully, several changes in the overstory and understory need to be thoroughly explored. In addition, abundant empirical knowledge is important for the development of management regulations that ensure the spatial and temporal continuity of forest cover through its natural regeneration (*Carrer, Soraruf & Lingua, 2013*).

Facilitating the natural regeneration of *L. principis-rupprechtii* plantations has always been a major challenge; indeed, only a few areas in northern China have well-regenerated *L. principis-rupprechtii* plantations. To identify the key factors controlling the natural regeneration of *L. principis-rupprechtii*, we first selected a study region with naturally regenerated *L. principis-rupprechtii* trees in Shanxi, China. In this region, the regeneration of *L. principis-rupprechtii* is restricted to a small area, suggesting that the conditions of this site might be conducive to the natural regeneration of this species. However, despite the presence of many adult trees, there were few regenerated seedlings. Thus, we studied three types of stands: (1) well-regenerated stands; (2) regenerated stands but with small numbers of regenerated plants; and (3) stands with no regeneration. By comparing the biotic and abiotic factors that affect regeneration in these three types of stands, we identified the main factors affecting the regeneration of larch. The results of this work provide important guidance for the management of later stages of natural regeneration to promote the sustainable development of *L. principis-rupprechtii* forest.

## Material and Methods

### Study area

The study sites were located at high elevations in the forest zone of Guandi Mountain in the middle of the Lvliang Mountains, west Shanxi Province, China (37°45′−37°55′N, 111°22′−111°33′E) (*Yang et al., 2017*). The annual mean temperature is 4.3 °C, and the mean annual precipitation along this gradient ranges from 600 mm to 822 mm (*Liu et al., 2007*). The region has a temperate continental climate, with long, cold and dry winters and short, warm and rainy summers, the coldest month is January (−10.2 °C), and the warmest month is July (17.5 °C); the growing season generally runs from June to September. The soil is classified as brown soil (Chinese classification) with an average thickness of 70–80 cm, including a 10-cm humus layer (*Yang et al., 2017*). The forest communities are primarily composed of *Picea*, *L. principis-rupprechtii*, *Betula, Populusdavidiana* and *Pinus tabulaeformis*. Shrub communities primarily consist of *Rosa xanthine*, *Hippophae rhamnoides, Vitex negundo* and *Heterophylla*. Herb communities include *Artemisia* and *Carex*.

### Community surveys

Community surveys were conducted in July 2019. A total of 18 plots (30 m ×30 m) were made, and each plot was divided into three forest types. Six plots had no regenerated larch seedlings, six plots had a few regenerated seedlings and six plots consisted of purely regenerated seedlings. Regeneration status was considered good if the number of seedlings > number saplings > number of trees and fair if the number of seedlings > number saplings. Regeneration was considered poor if the species was only present in the sapling stage and no seedlings were present or if there were no signs of regeneration despite the presence of adult trees (*Ballabha, Tiwari & Tiwari, 2013; Ali et al., 2019*).

The location of each plot (including elevation, longitude and latitude) was recorded using a navigation satellite time and ranging global position system receiver (TX35-S300, China) as well as the slope, slope position and slope aspect (*Li et al., 2018*). Variables measured in each plot were classified as stand structure data and included the following: (1) species, number, diameter at breast height (DBH), height and average crown; (2) identity and coverage of all shrub and herb species; (3) litter through the insertion of a metal ruler down to the soil surface at five points; and (4) tree stems, which were classified as either seedlings (height <50 cm), saplings (height > 50 cm and DBH(diameter at breast height) <5 cm) or adult trees (DBH > 5 cm) (*Carrer, Soraruf & Lingua, 2013; Muhamed et al., 2018*).

### Soil environmental surveys

Five soil samples was obtained with a “X” shaped collection scheme at depths of 0∼60 cm in each plot, because the soil layer below our study area primarily consists of stones. To mitigate the influence of precipitation on soil moisture, the soil samples were not collected within 2–3 days after rainfall. Wet and dry weighing methods were used to determine soil water content. The air-dried soil samples were then milled and sieved with a 2-mm screen in order to remove the debris and roots from the soil. The chemical properties were measured in the laboratory, i.e., soil organic matter (SOM) was determined using the K_2_Cr_2_O_7_ method; total nitrogen (TN) was measured using SKD-100 DigiPREP TKN Systems (Saacoo, Chongqing, China). Available nitrogen (AN) was extracted with KCl and quantified; on the other hand, total phosphorus (TP) was measured after extraction with HClO_4_-H_2_SO_4_. In addition, NaHCO_3_ solution was adopted to measure available phosphorus (AP) according to Olsen’s method and ammonium acetate solution was used to quantify available potassium (AK) (*Li et al., 2018*).

### Statistical analysis

Environmental gradients were identified through sequencing analysis using CANOCO (version 5.0). A preliminary detrended correspondence analysis for the regeneration of all community plots indicated that the gradient lengths were less than 3.0, which demonstrated that most regenerated plots performed linear responses to potential environmental variation, justifying the use of linear multivariate methods (*Li et al., 2018*). Based on this theory, the redundancy analysis (RDA) method was selected to identify the dominant environmental factors impacting on the community, implemented in the CANOCO 5.0 software package. The ranking scale focused on interspecies correlations, and the significance of data was evaluated via Monte Carlo test (*Chen, 2003*). Then, regeneration data was transformed through logarithmic conversion so that the effects of extreme values were mitigated (*Gazer, 2011*).

Species were indicated by the number and height of regeneration seedlings. Environmental factors were divided into four categories: topographic factors (elevation and slope), stand structural factors (density, average breast diameter, tree height and crown width), soil properties (soil organic matter, total nitrogen, total phosphorus, available phosphorus, available potassium and ammonia nitrogen) and litter (thickness and accumulation). All data for these factors were square-root transformed to ensure that variances were homogeneous before statistical analyses.

Using ANOVA method, the logarithmic values after transformation were calculated. Duncan’s multiple range test (*P* < 0.01 or *P* < 0.05) was used to compare differences in topographic factors, stand structural factors, soil properties and litter. And SPSS 22.0 software was adopted to analyze descriptive statistical parameters and significance of all the indicators.

## Results

**Figure 1:**
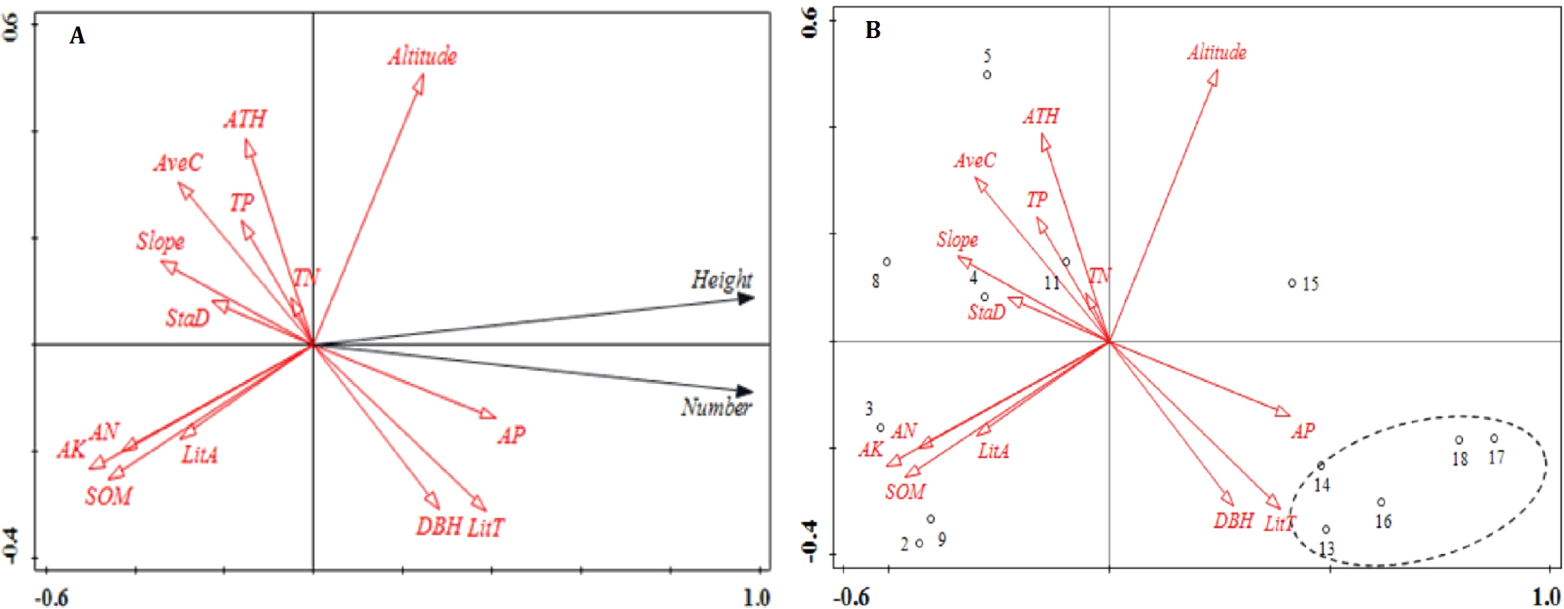
Ordination diagram of the results of the RDA analysis of stand structure, soil nutrients, litter and topographic variables. (A) The relationship between number, height, and impact factors; (B) the relationship between plot distribution and impact factors. The direction of the arrows corresponds to the directions of the changes, and the lengths of the arrows indicate the extent to which a factor impacts regeneration. Abbreviations of stand structure, soil nutrients and litter variables are as follows: StaD, stand density; DBH, average diameter at breast height; ATH, average tree height; Avec, average crown; SOM, soil organic matter; TN, total nitrogen ; AP, available phosphorus; AK, available potassium; TP, total phosphorus; AN, ammonia nitrogen; TitT, litter thickness and TitA, litter accumulation.

RDA can be used to distinguish the relative contribution of each soil nutrient, stand structure, litter and topographical variable on regeneration, and to assess the correlations between variables and multivariable data, by determining the best predictor of vegetation regeneration based on statistical theory. The RDA ordination diagram is shown in Fig. 1. The correlations between corresponding variables are indicated by the cosine values between the environmental variables in the figure. A positive cosine value represents a positive correlation between the respective variables while a negative cosine value indicates a negative correlation.

The results of the RDA showed that 87.2% of the variation in regeneration was explained by the first axis (Fig. 1). Available P, litter thickness, DBH and altitude were positively correlated with the number of regenerated plants. Litter thickness had the strongest effect on the number of regenerated plants while altitude had the weakest effect. The factors that were negatively correlated with the number of regenerated plants included soil organic matter, available K, ammonia nitrogen, slope, average crown and average tree height; the variable with the strongest negative correlation with the number of regenerated plants was available K. Aside from plot 15, plots 13–18 showed consistent patterns. Specifically, these plots were areas that showed the greatest degrees of regeneration. Available P, litter thickness and DBH were also correlated with regeneration.

The RDA1 axis was strongly correlated with available K, soil organic matter, available P, litter thickness and ammonia nitrogen (Table 1). Total N showed the lowest correlation coefficient with RDA1 (−0.060) followed by average tree height and total P (Table 1). Altitude was the variable most strongly correlated with RDA2 (0.528); all other variables showed lower correlations (Table 1).

**Table 1:** Correlation coefficients for environmental variables for RDA 1 and RDA 2.

Factor	RDA1	RDA2
Altitude	0.200	0.528
Slope	−0.356	0.126
Litter thickness	0.414	−0.277
Litter accumulation	−0.282	−0.203
Average DBH	0.309	−0.282
Average tree height	−0.187	0.372
Average crown	−0.330	0.278
Stand density	−0.235	0.060
Soil organic matter	−0.436	−0.295
Total N	−0.060	0.085
Total P	−0.183	0.216
Ammonia nitrogen	−0.412	−0.238
Available P	0.421	−0.102
Available K	−0.481	−0.278

In the RDA analysis, seven factors that had a contribution rate greater than 10%–AK > SOM > AP > LitT > AN > Slope > AveC—which was consistent with the correlation analysis of the factors (Table 2).

**Table 2:** Percent variance explained of the extent of regeneration by each environmental factor.

Factor	Explains %	Pseudo-F	*P*
AK	22.5	4.7	0.058
SOM	18.6	3.6	0.086
AP	17.3	3.3	0.068
LitT	16.7	3.2	0.098
AN	16.6	3.2	0.082
Slope	12.4	2.3	0.124
AveC	10.6	1.9	0.194
DBH	9.3	1.6	0.220
LitA	7.8	1.3	0.276
StaD	5.4	0.9	0.338
Altitude	3.9	0.6	0.474
ATH	3.4	0.6	0.444
TP	3.3	0.5	0.472
TN	0.4	<0.1	0.824

Table 3 shows the correlations between soil nutrients, stand structure, litter and topographic factors obtained through the RDA. The extent of regeneration was positively correlated with tree regeneration height (0.983), available P (0.416) and litter thickness (0.409). Available K (−0.475), soil organic matter (−0.431) and available N (−0.407) were negatively correlated with the extent of regeneration. Altitude was positively correlated with stand density (0.563) but was negatively correlated with DBH (−0.734). The slopes of the plots revealed a positive correlation between average tree height (0.831) and stand density (0.760). These results suggested that topographic factors were positively correlated with stand density. In addition, litter thickness was positively correlated with litter accumulation (0.564). Litter accumulation was positively correlated with available N (0.665), total P (0.562) and soil organic matter (0.460).

**Table 3:** Correlation analysis of several environmental factors.

	Number	Height	Altitude	Slope	LitT	LitA	DBH	ATH	AveC	StaD	SOM	TN	TP	AN	AP	AK
Number	1															
Height	.983^**^	1														
Altitude	.194	.286	1													
Slope	−.352	−.325	.196	1												
LitT	.409	.355	.310	.015	1											
LitA	−.279	−.311	.012	−.201	.564^*^	1										
DBH	.305	.251	-.734^**^	−.178	−.240	−.392	1									
ATH	−.185	−.116	.373	.831^**^	−.021	−.291	−.143	1								
AveC	−.326	−.272	−.258	−.266	−.217	.222	.109	−.067	1							
StaD	−.227	−.220	.563^*^	.760^**^	.224	−.032	-.588^*^	.533^*^	-.607^**^	1						
SOM	−.431	-.478^*^	−.259	−.170	.050	.460	−.024	−.207	.519^*^	−.225	1					
TN	−.059	−.043	.069	−.162	−.046	.067	−.102	−.296	−.080	.015	−.320	1				
TP	−.181	−.140	.383	.011	.344	.562^*^	-.607^**^	.043	.067	.177	−.250	.321	1			
AN	−.407	−.444	−.122	−.278	.162	.665^**^	−.227	−.313	.536^*^	−.213	.896^**^	−.164	.038	1		
AP	.416	.392	−.203	−.103	.210	−.132	.450	−.072	.161	−.329	−.177	.036	−.045	−.251	1	
AK	-.475^*^	-.518 ^∗^	−.045	.427	.082	.271	−.271	.246	−.127	.393	.260	.148	−.002	.190	−.277	1

**Notes.**

* showed significant difference at the 0.05 level.

** showed significant difference at the 0.01 level.

**Figure 2:**
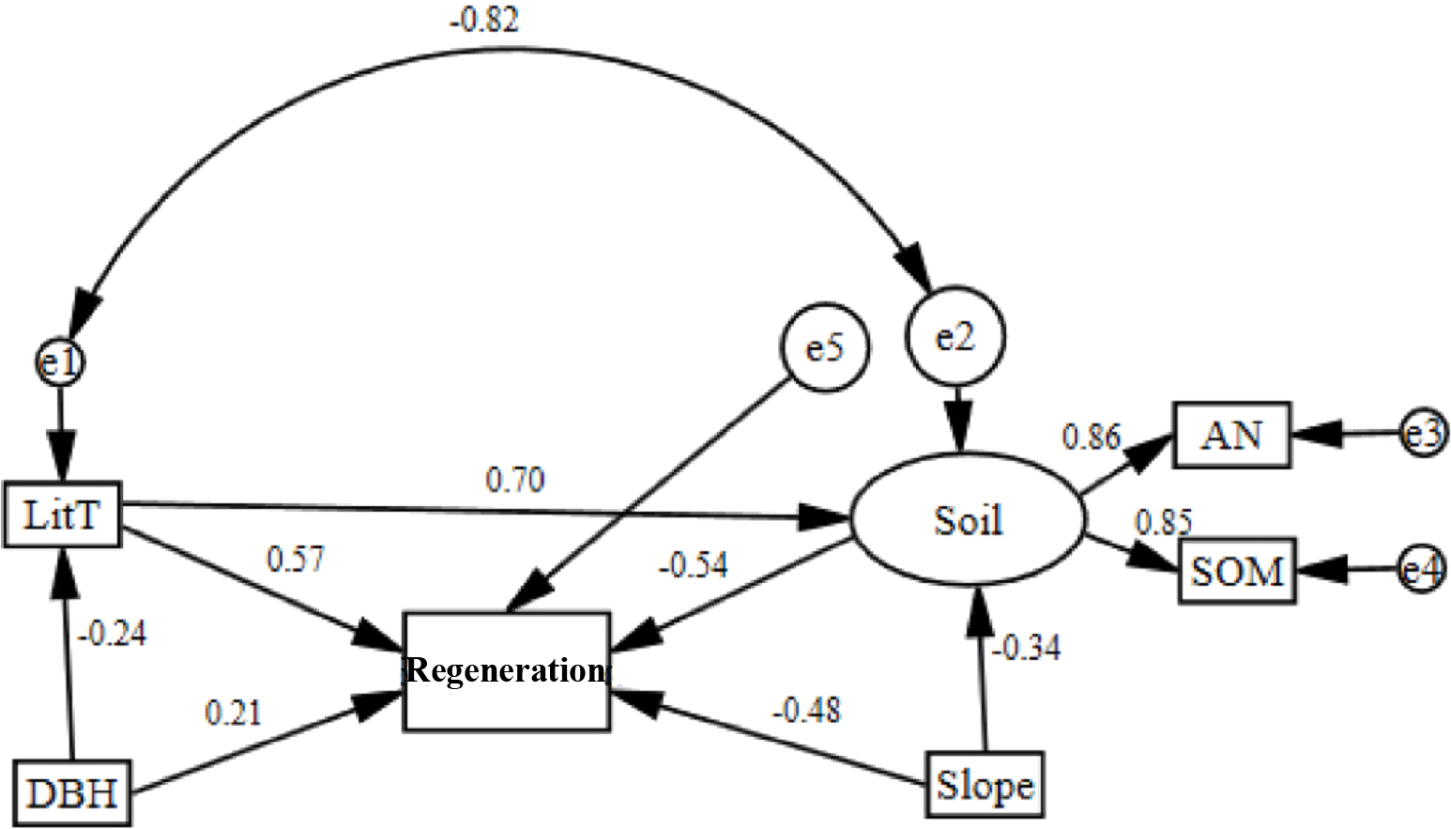
Corrected structural equation model with standardized path coefficients between influence factors and regeneration.

Based on the RDA, a structural equation model (SEM) was established for all factors that had a contribution rate greater than 10% and were highly relevant (Table 2; Table 3). According to the model-fitting criteria, the removal of factors did not have a significant effect on regeneration. The model was then corrected several times. Finally, the five factors most closely related to regeneration were selected to construct the SEM. The final model is shown in Fig. 2. The model had a high degree of fit (*P* = 0.339, *χ*^2^/*df* = 1.236). The incremental fit index (IFI) and comparative fit index (CFI) were 0.987 and 0.984, respectively, while the standardized root mean square residual (SRMR) and the root mean square error of approximation (RMSEA) were 0.078 and 0.080, respectively. All of the fit indexes were high (Table 4).

**Table 4:** Fitting of the SEM parameters for the most important factors affecting regeneration.

Baseline Comparisons					Simple with moderate index			
*χ*^2^/*df*	*p*-value	GFI	AGFI	NFI	SRMR	IFI	CFI	RMSEA
1.136	0.339	0.916	0.905	0.903	0.078	0.987	0.984	0.080

Soil nutrients and regeneration were negatively correlated and had a path coefficient of −0.54 (Fig. 2). Soil nutrients were positively correlated with ammonia nitrogen and soil organic matter, the path coefficients of which were 0.86 and 0.85, respectively. Regeneration was negatively correlated with slope and had a path coefficient of −0.34. Litter thickness and DBH were positively correlated with regeneration and had path coefficients of 0.57 and 0.21.

## Discussion

This analysis of one of the most comprehensive regional datasets on the regeneration of *L. principis-rupprechtii* plantations suggested that the slope, DBH, litter thickness, ammonia nitrogen and soil organic matter were the most significant factors affecting regeneration.

### Effects of topography on regeneration

Micro-topographic factors, such as slope, greatly impact plant community structure and patterns of regeneration. The ordination results indicated that the slope was the main terrain factor affecting regeneration (*Wang et al., 2016*). The slope was positively related to aboveground biomass and herb coverage but negatively related to regeneration and stand density. However, the effects of topographic factors (−0.48) on regeneration were less significant compared with soil factors (−0.54). In our study area, the difference between the maximum and minimum elevations in the sampling plots was 124 m, which is not likely sufficient for causing significant variations in thermal conditions and thus affecting regeneration (*Liu et al., 2012*). Topographic factors affected both vegetation and soil nutrients. Topography can partially influence the accumulation and export of soil nutrients and thereby indirectly impact the distribution of plants. For example, previous studies have indicated that topographic changes can have strong effects on soil physical and chemical properties and soil moisture characteristics (*Sariyildiz, Anderson & Kucuk, 2005; Dessalegn et al., 2014*). Here, regeneration was negatively correlated with slope, suggesting that it is easier for seeds to accumulate on gentle slopes.

### Effects of stand structure on regeneration

The relative contributions of different factors also varied depending on the life history stages of regenerated seedlings. The height structure of the regenerated seedlings is an important factor affecting the regeneration of trees, and reflects the status of stand regeneration (*Kayes & Tinker, 2012*). Stand structure factors also affect restoration and play important roles in regulating the natural regeneration of forest (Chen & Cao, 2014). The RDA analysis and SEM all showed that tree DBH was positively correlated with seedling regeneration. Parent trees with larger DBH generally obtain more resources to support their growth and occupation of a dominant position in the forest stand (Qian et al., 2014). The number of seeds produced by robust parent trees is higher in both quantity and quality and might explain the high number of regenerated seedlings. There was a positive correlation between stand crown and regeneration, but the strength of this correlation was relatively weak. Overall, the relationship between larch regeneration, DBH and stand density was highly consistent in our study site and is consistent with the results of other studies (*Hébert, Krause & Plourde, 2016; Ali et al., 2019*).

### Effects of shrubs, herbs and litter on regeneration

Undergrowth vegetation is a critically important factor affecting forest ecosystems (*Nilsson & Wardle, 2005*), as it has an inhibitory effect on forest regeneration and, in turn, on forest dynamics (*Montti & Campanello, 2011*). However, shrubs were generally few and scattered in our plots, suggesting that they had little effect on stand regeneration. In regeneration plots, saplings grew well and were distributed in clusters, which inhibited the growth of herbs. Herbs did not affect the regeneration of seedlings, given that *L. principis-rupprechtii* is a shade-tolerant plant (*Wagner et al., 2010; Lombaerde et al., 2019*). Indeed, the shade provided by herbs protects the growth of the seedlings. Eventually, seedlings suppress the growth of herbs, which explains the decrease in herbs observed as a consequence of canopy closure (*Soucy, Lussier & Lavoie, 2012*).

*L. principis-rupprechtii* seeds were approximately 1–2 mm in length and were primarily concentrated in the litter layer. The heat preservation and water retention capacity of the litter layer provided prime conditions for seed germination. Many studies have shown that seed survival rate is significantly higher in litter than in bare habitats (Ibáñez & Schupp, 2002; Muhamed et al., 2018). This study also showed that litter thickness was closely related to the regeneration of larch, with an RDA contribution rate of 16.7% and an SEM path coefficient of 0.57. Other studies have shown that litter inhibits seed germination; for example, regeneration seedlings often failed to obtain sufficient nutrients given that the radicle could not reach the soil, resulting in increased mortality in regeneration seedlings (*Nakagawa, Kurahashi & Hogetsu, 2003*). The continuous accumulation would also impede the natural regeneration of vegetation, primarily via physical barriers, chemical effects, infestations of animals and microbial pathogenic effects. However, litter thickness was positively correlated with regeneration. This correlation might be explained by the moderate litter thickness in the study area, which did not accumulate and affect seed germination and seedling growth. However, this correlation also suggested that the litter is decomposed at an adequate rate and reflects an overall healthy ecological cycle in the study area. Litter layers of this thickness provided sufficient water for the seeds, facilitated the return of nutrients to the soil, promoted the growth of newer seedlings and also provided some protection for the seeds by reducing avian damage. Thus, this study suggests that the litter thickness in these plots represent thicknesses that would be ideal for stands of *L. principis-rupprechtii* in this region. In stands with thicker litter layers, litter could be managed to both facilitate the natural regeneration of the stand as well as nutrient cycling in the soil.

### Relationship between soil nutrients and regeneration

Forest regeneration has a clear, predictive effect on soil nutrient contents (*Fu, Qi & Chang, 2015*); soil variables, such as AP, AK, ammonia nitrogen and soil organic matter; and the growth, reconstruction and development of vegetation (Qian et al., 2014). According to the SEM, ammonia nitrogen and soil organic matter made the largest contributions to explaining regeneration compared with other variables relating to soil properties (*Wang et al., 2016*). Because a large amount of litter is produced in the soil every year, the decomposition of litter increases soil organic matter. Consistent with this expectation, soil organic matter was positively correlated with average crown and negatively correlated with the height of regenerated seedlings; and high, but not significant, correlations were observed between average crown and seedling height. Thus, plants might increase soil organic matter content.

Similar to previous studies on forest regeneration (*Chen, 2003; Bünemann et al., 2004*), AP was one of the most important variables affecting variation in species richness, and its response curve was consistent with the response curve for regeneration. Ammonia nitrogen had the strongest effect on changes in species composition at our study sites, but the number of species varied little relative to variation in ammonia nitrogen. For instance, the number of species was at its maximum when ammonia nitrogen was relatively low (*Liu et al., 2011*). The richness and quantity of species varied in the RDA, demonstrating that both the diversity and numbers of species—specifically the ‘regeneration quantity’ and ‘valuable indigenous species’—were insufficient and that further restoration and cultivation are needed. High contents of ammonia nitrogen and soil organic matter inhibit regeneration; however, regenerated seedlings might have a higher consumption of ammonia nitrogen and soil organic matter (*Xu et al., 2018*).

### Management implications

The most important factors affecting the regeneration of *L. principis-rupprechtii* plantations included ammonia nitrogen, soil organic matter, litter thickness, DBH and slope. These factors are also important in other systems (*Puhlick, Laughlin & Moore, 2012*). Thus, measurements of these variables could provide forest managers a means of assessing the state of regeneration in a given stand. The removal of litter to reduce its thickness could improve seed germination and promote regeneration. The removal of unhealthy trees in the forest could increase the space available to mother trees and promote their growth. However, increases in light and microbial activity accelerate the decomposition of litter, reducing its thickness and increasing soil organic matter. Overall, appropriate management actions can promote the robust regeneration of forest stands.

## Conclusions

A comprehensive understanding of how environmental factors affect tree seedlings is necessary for ensuring the success of the natural regeneration of plantations. Here, we used RDA and SEM to evaluate the relationships between four types of environmental factors and regeneration of *L. principis-rupprechtii* plantations. There was much variation in the relationships of topographic factors, stand structural factors, soil properties and litter with tree regeneration. Slope and soil properties were negatively correlated with regeneration; in contrast, DBH and litter thickness were positively correlated with regeneration. Furthermore, litter thickness was the most significant factor affecting tree regeneration (coefficient of 0.57), and soil was the second most important factor (coefficient of −0.54). Among the indicators of soil properties, ammonia nitrogen and soil organic matter were considered the most important factors affecting the success of regeneration. Therefore, interventions to enhance the aforementioned significant factors would provide a robust means for managers to stimulate tree regeneration and maintain the health and sustainability of plantations.

##  Supplemental Information

10.7717/peerj.9339/supp-1 Supplemental Information 1 Impact factor data

## Additional Information and Declarations

### Field Study Permissions

The following information was supplied relating to field study approvals (i.e., approving body and any reference numbers): Field experiments were conducted at the base of Shanxi Agricultural University for teaching practice.

### Data Availability

The following information was supplied regarding data availability: The raw measurements are available in the Supplementary File.
